# Chinese University Students’ Perceptions of Facilitation Strategies, Learning Motivation, and Satisfaction in Cloud-Based Virtual Classrooms

**DOI:** 10.3389/fpsyg.2021.801191

**Published:** 2021-12-14

**Authors:** Rong Wang, Jiying Han, Chao Gao, Chuanyong Liu

**Affiliations:** ^1^Department of Physiology and Pathophysiology, School of Basic Medical Sciences, Shandong University, Jinan, China; ^2^School of Foreign Languages and Literature, Shandong University, Jinan, China; ^3^School of Foreign Languages, Shandong Women’s University, Jinan, China

**Keywords:** facilitation strategies, student satisfaction, cloud-based virtual classroom, mainland China, student learning motivation, facilitation strategies, learning motivation, satisfaction

## Abstract

This study investigated university students’ perceptions of facilitation strategies, learning motivation, and satisfaction, and the relationships between them in a cloud-based virtual classroom in mainland China. The results of an online questionnaire survey from a sample of 7,210 university students showed that students perceived high levels of facilitation strategies, learning motivation, and satisfaction. Students’ demographic characteristics, such as discipline, university type, gender, and grade, did not significantly affect their perceptions of facilitation strategies and learning outcomes. Instructor-student interaction and instructor innovation were positively related to student learning motivation and satisfaction whereas the relationships between student interaction and learning motivation and satisfaction were weak and had no practical meaning. The findings of this study have implications for creating more effective synchronous online learning environments and achieving desirable learning outcomes.

## Introduction

The learning environment is a key determinant of students’ cognitive, affective, and behavioral outcomes (Allen and Fraser, 2007). As one of the most important aspects of the learning environment, the role of instructors in students’ learning success is well documented (Lim and Morris, 2009; Wang R. et al., 2021). The instructional strategies adopted by facilitators are positively related to students’ cognitive and psychological development outcomes (Lazowski and Hulleman, 2015). Recently, the global COVID-19 pandemic has given rise to an increasing number of studies exploring students’ learning effectiveness in synchronous online learning environments (e.g., Iglesias-Pradas et al., 2021; Tang et al., 2021). Preliminary studies have indicated that the availability of cloud services in the synchronous online learning environment provides more efficient technological support for interactions between students and instructors (e.g., Baanqud et al., 2020). Among these interactions, instructors’ timely response and feedback to questions and assignments are the most effective facilitation strategies (Martin et al., 2018, 2019). However, while a majority of studies on online learning environments have been conducted in asynchronous or blended learning environments (e.g., Lim and Morris, 2009; Cho and Cho, 2014), students’ perceptions of how their instructors’ facilitation strategies influence their learning outcomes in a synchronous online learning environment remain underexplored.

In mainland China, the outbreak of coronavirus disease 2019 (COVID-19) has resulted in the cancelation of all face-to-face teaching activities at all educational levels. At the request of China’s Ministry of Education, schools and universities have been delivering their teaching activities through synchronous virtual classrooms on an unprecedentedly large since February 2020, and are making good use of online learning technologies. Within this changed learning environment, how do university students perceive the various facilitation strategies adopted by their instructors? How do these facilitation strategies influence students’ affective outcomes? The present study aimed to address these questions by exploring the relationships between university students’ perceptions of facilitation strategies adopted by their instructors and students’ learning outcomes in cloud-based virtual classrooms in mainland China.

## Literature Review

### Facilitation Strategies as an Environmental Factor

The learning environment has attracted considerable interest from educational researchers in the past few decades, and it has been shown to be a significant determinant of students’ cognitive and affective outcomes (Eom et al., 2006; Al-Samarraie and Saeed, 2018). The role of instructors, one of the key elements of the learning environment, has been acknowledged as a significant determinant of student learning (Martin et al., 2019). Instructors can be effective facilitators of students’ learning, and the various strategies they adopt to motivate and encourage students’ effective online learning are facilitation strategies. There is extensive evidence confirming that facilitation strategies are effective in supporting students’ learning processes and outcomes (e.g., Martin et al., 2019; Xu et al., 2020). However, as a majority of these studies were conducted in asynchronous online or blended learning environments, there is a paucity of research on the relationship between instructors’ scaffolding and students’ learning in synchronous online learning environments (Kuo et al., 2014). As instructors have been found to adopt different teaching strategies for different educational contexts (Lindblom-Ylänne et al., 2006), it is important to investigate the facilitation strategies of university teachers in the cloud-based virtual classroom following the outbreak of COVID-19 in China.

In online learning environments, facilitation strategies refer to the diverse strategies adopted by the instructor to motivate and encourage students to learn more effectively (Martin et al., 2018). Instructor scaffolding for interaction, which refers to instructors’ implementation of strategies to promote learner-instructor and learner-learner interaction, has been identified as an essential element of students’ learning success and a driving force for promoting their motivation and learning outcomes (Mamun et al., 2020). A recent study indicated that among four types of facilitation strategies, timely responses to questions and feedback on assignments were most effective in facilitating students’ learning, leading to the need for teaching innovation, especially creative methods for providing timely feedback (Martin et al., 2018). Instructor innovation is a significant facilitation strategy for effective online teaching and learning (Al-Samarraie and Saeed, 2018). The patterns of innovation that instructors adopt include different teaching methods from those in face-to-face courses, newly designed online learning activities to get students involved and different learning tasks (Fraser et al., 1986). Therefore, with the purpose of exploring the influence of facilitation strategies on students’ learning in cloud-based virtual classrooms, this study measured students’ perceptions of facilitation strategies in terms of student interaction, instructor-student interaction, and instructor innovation.

### Learning Motivation in Online Learning Environments

A considerable number of studies have revealed the relationship between students’ perceptions of the learning environment and their affective learning outcomes, and students’ learning motivation is the most-often researched indicator of their affective outcomes (Cho and Cho, 2014; Law et al., 2019). Learning motivation, as one of the important variables in emotion-based studies (Wang Y. et al., 2021), refers to the internal (intrinsic) and external (extrinsic) forces that give students the power to learn effectively (Keller, 2007), and involves a learner’s feelings and emotions during the learning process (Wlodkowski, 1999). Despite various motivational theories, self-efficacy, a powerful mechanism in explaining human motivation, has received much attention in online learning environments because of its significant effect on learning outcomes through dynamic interactions with environmental and behavioral factors (Bandura, 1997, 2012). Self-efficacy refers to a person’s belief in his or her capabilities to accomplish a learning or performance task in an educational setting (Bandura, 1986). It is well acknowledged that self-efficacy is positively connected with online learning outcomes (Honicke and Broadbent, 2016). Bandura (1997) identified four sources of self-efficacy: performance accomplishments, vicarious experience, social persuasion, and physiological and emotional states, and previous studies have shown that students’ self-efficacy in online learning may be enhanced by the support connected with the four sources (Abbitt and Klett, 2007; Han et al., 2021).

As a context-specific and task-dependent concept (Schunk and Pajares, 2002), self-efficacy in online learning environments has been categorized into academic self-efficacy (ASE) and Internet or computer self-efficacy (ISE) (Tsai et al., 2011). However, previous studies have found that the level of confidence in using Internet-based technologies or ISE did not significantly contribute to students’ learning satisfaction (Kuo et al., 2014). However, it is still unknown whether learners’ perceptions of ASE can predict their online learning success (Tsai et al., 2011). As most studies have been conducted in asynchronous or blended online learning settings, researchers have indicated the need to develop relevant strategies for enhancing self-efficacy in the context of synchronous online learning for better educational outcomes (Lazowski and Hulleman, 2015; Martin et al., 2018). Therefore, further investigation of students’ self-efficacy and its relationship with various facilitation strategies in synchronous online learning environments will help to fill these research gaps.

Affect/emotion is an element of learner motivation during the learning experience (Lim and Kim, 2003). The established relationship between learners’ emotions and their academic performance in online learning environments means that emotions are important considerations for instructors making instructional decisions in online teaching (Fatahi, 2019). Studies exploring the relationships between facilitation strategies and student affect/emotion have revealed a positive relationship between instructor presence, i.e., the specific actions and behaviors of instructors, and student emotion/affect in asynchronous and blended online learning environments (Law et al., 2019; Martin et al., 2019). However, the relationship between online facilitation strategies, including scaffolding and innovation and students’ affect/emotion in synchronous online learning environments, is worth further exploration.

### Student Satisfaction in Online Learning Environments

Student satisfaction reflects students’ feelings, attitudes, and hopes about the quality of the learning environment (Wu et al., 2010). Student satisfaction reflects the learning outcomes that occur between student and instructor (Thurmond, 2003; Yunusa and Umar, 2021) and is a widely used indicator of students’ attitudes toward the online learning process (Graham and Scarborough, 2001). Many studies have found positive relationships between students’ perceptions of facilitation strategies, student satisfaction, and learning achievement in asynchronous and blended learning environments. For example, instructor presence has been found to be effective in helping students to overcome their feelings of isolation and the lack of support from their instructor and peers (Cho and Summers, 2012), and to increase student satisfaction (Richardson et al., 2017). However, although a number of facilitation strategies have been shown to be positively related to student satisfaction (Wu, 2002), very few facilitation strategies have been examined in synchronous learning environments (Martin and Parker, 2014). Given the widespread use of cloud-based virtual classrooms in synchronous learning environments, studies exploring the relationships between various facilitation strategies and student satisfaction could help to inform responses to the current changes and challenges in higher education.

## Purpose of This Study

This study aimed to address the following two research questions based on the literature: (1) What are university students’ perceptions of the facilitation strategies adopted by instructors in cloud-based virtual classrooms in mainland China? (2) What are the relationships between students’ perceptions of facilitation strategies, affective learning outcomes (self-efficacy, affect, and emotion), and satisfaction?

## Methodology

### Participants

The participants were 7,210 undergraduate students (62.20% female, 37.80% male) with different disciplinary backgrounds. Of these, 5,045 (70%) were from a national key university and 2,165 (30%) from a local provincial university in Shandong, a province in eastern China. An online questionnaire survey was conducted in April 2020, three months after the start of synchronous online learning. With a clear instruction of the purpose of the study, all participants were invited to fill in the questions of the online survey on an anonymous and voluntary basis. The demographic characteristics of the study sample were presented in Table 1.

**TABLE 1:** The demographic characteristics of the sample (*N* = 7,210).

Category		Total and *N* = 7,210		Key university and *N* = 5,045		Local university and *N* = 2,165	
		*n*	%	*n*	%	*n*	%
Gender	Female	4,484	62.2	2,851	56.5	1,633	75.4
	Male	2,726	37.8	2,194	43.5	532	24.6
Grade	Freshman	3,837	53.2	2,401	47.6	1,436	66.3
	Sophomore	1,782	24.7	1,363	27.0	419	19.4
	Junior	1,266	17.6	960	19.0	306	14.1
	Senior	325	4.5	361	6.4	4	0.2
Discipline	Social science and humanities	4,018	55.7	2,443	48.4	1,575	72.8
	Science	1,348	18.7	839	16.7	509	23.5
	Technology	1,631	22.6	1,550	30.7	81	3.7
	Medicine	213	3.0	213	4.2	0	0

### Instruments

The questionnaire comprised two sections. The first section included demographic information such as gender, grade, discipline, and university type. The second section consisted of three self-report instruments to measure facilitation strategies, online learning motivation, and student satisfaction. A 5-point Likert-type response scale was used for all three self-report instruments, where 1 denoted “strongly disagree” and 5 “strongly agree.”

#### Facilitation Strategies

Students’ perceptions of facilitation strategies were measured in three dimensions: instructor-student interaction, student interaction, and instructor innovation. Measures were adapted from the Classroom Environment Scale (Fraser and Fisher, 1983; Moos and Trickett, 1987), the College and University Classroom Environment Inventory (Fraser et al., 1986), and the study by Johnson et al. (2000) to better capture online learning environments. A total of ten items were used to measure instructor-student interaction, (three items, e.g., “The instructors encouraged me to become actively involved in the discussions,”) student interaction (three items, e.g., “The online course enables interactive communication among students,”) and instructor innovation (four items, e.g., “The instructors use various and innovative teaching methods in the online course”).

#### Learning Motivation

To assess learning motivation, seven items in two subscales (self-efficacy and affect/emotion) were adapted from the Learning Motivation Questionnaire (Lim and Kim, 2003). The items were considered to reflect self-efficacy (three items, e.g., “I utilize effective study skills in learning new concepts”) and affect/emotion (four items, e.g., “Completing online course assignments gives me a feeling of accomplishment”).

#### Student Satisfaction

To measure student satisfaction, five items were adapted from the study by Lee et al. (2011). The original questionnaire had five items; however, the last two items were revised to better capture the perception of Chinese university students’ perceptions of the online course quality. An example item is “I felt satisfied with the online course.”

### Data Analysis

Confirmatory factor analysis (CFA) was conducted using AMOS 22.0 to examine the construct validity of the instruments used in this study. Cronbach’s α reliability coefficients were computed by SPSS 22.0 to examine the reliability of the subscales. A repeated measures one-way ANOVA was conducted to determine whether there were significant differences between the mean scores of the subscales. Multivariate analysis of variance (MANOVA) was used to identify whether the students’ perceptions of facilitation strategies, affect/emotion, self-efficacy, and satisfaction varied across gender, discipline, and grade. A full structural equation model (SEM) was then developed to examine the relationships between the independent environmental variables (facilitation strategies) and the dependent variables (affect/emotion, efficacy, and satisfaction) using AMOS 22.0. All results were explained in terms of effect size according to Gignac and Szodorai’s (2016) suggested guidelines (small = 0.10– < 0.20, medium = 0.20–< 0.30, large ≥ 0.30).

## Results

### Construct Validity and Reliability

We first conducted a series of CFA to examine the construct validity of each instrument using AMOS 22.0. The goodness-of-fit indices used in this study were χ^2^ statistics, degrees of freedom (*df*), comparative fit index (CFI) > 0.90, Tracker-Lewis index (TLI) > 0.90, and root-mean-square error of approximation (RMSEA) < 0.08. As shown in Table 2, the three-factor measurement model of the facilitation strategies fitted the data (χ^2^ = 19.74, *df* = 51, *p* < 0.01, CFI = 0.99, TLI = 0.98, RMSEA = 0.051). The factor loadings of all items ranged from 0.60 to 0.80, and the Cronbach’s alpha coefficients for the three sub-scales were 0.91 (instructor-student interaction), 0.90 (student interaction) and 0.91 (instructor innovation), suggesting good internal consistency for each sub-scale.

**TABLE 2:** CFA results for the scales.

Scale	χ^2^	*df*	*p*	CFI	TLI	RMSEA
Facilitation strategies	19.74	51	0.00	0.99	0.98	0.051
Learning motivation	45.13	19	0.00	0.98	0.97	0.08
Student satisfaction	115.74	5	0.00	0.98	0.96	0.06

The CFA results for learning motivation shown in Table 2 indicated an adequate model fit (χ^2^ = 45.13, *df* = 19, *p* < 0.01, CFI = 0.98, TLI = 0.97, RMSEA = 0.08). The factor loadings of the learning motivation items ranged from 0.42 to 0.48. The CFA results of student satisfaction indicated a good model fit (χ^2^ = 115.74, *df* = 5, *p* < 0.01, CFI = 0.98, TLI = 0.96, RMSEA = 0.06). The factor loadings of all items ranged from 0.53 to 0.87. The Cronbach’s alpha coefficients were 0.90 (affect/emotion), 0.88 (general self-efficacy) and 0.93 (student satisfaction).

### Descriptive Statistics and Correlations

We then calculated the descriptive statistics (*M* and *SD*) and Pearson correlations matrix for all factors by SPSS 22.0. Table 3 presents the descriptive statistics and correlation matrix. The mean scores for all subscales were higher than the midpoint of 3, indicating that students perceived high levels of online learning outcomes and facilitation strategies. Among the subscales, the mean scores of the three facilitation strategies were above 3.80, and instructor-student interaction had the highest score (*M* = 3.86, *SD* = 1.03). The correlation matrix indicated that the three facilitation strategies were positively and significantly correlated with students’ affect/emotion, self-efficacy, and satisfaction.

**TABLE 3:** Descriptive statistics, reliabilities and correlations of the factors (*N* = 7,210).

	Instructor-student interaction	Student interaction	Instructor innovation	Affect/emotion	Efficacy	Student satisfaction
Instructor-student interaction	(0.91)					
Student interaction	0.69**	(0.90)				
Instructor innovation	0.75**	0.72**	(0.91)			
Affect/emotion	0.67**	0.58**	0.64**	(0.90)		
Efficacy	0.66**	0.61**	0.65**	0.78**	(0.88)	
Student satisfaction	0.66**	0.58**	0.63**	0.75**	0.75**	(0.93)
*Mean*	3.86	3.85	3.80	3.61	3.45	3.34
*Standard Deviation*	1.03	1.04	1.03	1.11	1.11	1.16

**p < 0.01; Cronbach’s α coefficients in parentheses along the diagonal.

### Inferential Analysis

To examine the university students’ perceptions of facilitation strategies, affective learning outcomes (affect/emotion and efficacy), and online satisfaction, we also conducted repeated measures of one-way ANOVA with SPSS 22.0 to determine whether there were significant differences between the mean scores of the subscales. We chose the Greenhouse-Geisser correction test to compare the differences because the sphericity of variance assumption was violated. The results indicated a significant difference between the mean scores of the three facilitation strategies [*F*_(1.95, 14077.43)_ = 47.78, *p* < 0.001] and between learning motivation and satisfaction [*F*_(1.94, 13979.56)_ = 705.98, *p* < 0.001]. *Post hoc* Bonferroni tests indicated that the mean scores for instructor-student interaction (*M* = 3.86, *SD* = 1.03) and student interaction (*M* = 3.85, *SD* = 1.04) were significantly higher than that of instructor innovation (*M* = 3.80, *SD* = 1.03). There were significant differences between the mean scores for affect/emotion (*M* = 3.61, *SD* = 1.11), self-efficacy (*M* = 3.45, *SD* = 1.11), and satisfaction (*M* = 3.34, *SD* = 1.16).

MANOVA was used to examine whether students’ perceptions of facilitation strategies, online learning motivation, and satisfaction differed significantly among those with different demographic characteristics: gender, grade, discipline, and university type. We found a significant main effect of discipline [*F*_(15_, _21612)_ = 5.42, *p* < 0.01] on each of these variables except for instructor-student interaction, and a significant main effect of university type [*F*_(3_, _7206)_ = 54.94, *p* < 0.001] on each variable except for instructor innovation. However, the global model effect sizes of discipline (η^2^ = 0.004) and university type (η^2^ = 0.02) were very small (< 0.10) and had no practical significance. The main effects of gender and grade were not significant.

### Structural Equation Model Analysis

**FIGURE 1:**
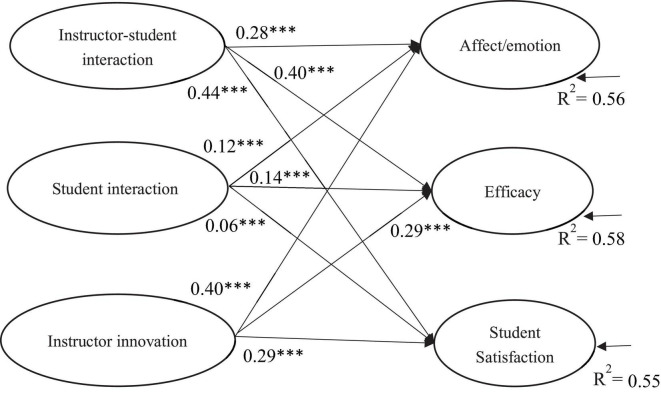
SEM model results showing significant regression paths. ^***^p < 0.001; Goodness-of-fit indices: χ^2^ = 8081.57, *df* = 262, *p* < 0.001, CFI = 0.95, TLI = 0.94, RMSEA = 0.064.

To address the second question, we performed SEM using AMOS 22.0 to explore the relationships between facilitation strategies, affect/emotion, efficacy, and satisfaction. The model was based on the hypothesis of correlations between the independent environmental variables (facilitation strategies) and the dependent variables (affect/emotion, efficacy, and satisfaction). The SEM results (see Figure 1) indicated that the model fitted the data well (χ^2^ = 8081.57, *df* = 262, *p* < 0.001, CFI = 0.95, TLI = 0.94, RMSEA = 0.064), and explained 0.56 (affect/emotion), 0.58 (efficacy) and 0.55 (satisfaction) of their variances. The results revealed that instructor-student interaction and instructor innovation were positively related to affect/emotion, efficacy and satisfaction, and the effect sizes of these associations were medium and large (> 0.20). However, although student interaction was significantly related to affect/emotion (β = 0.12, *p* < 0.001), efficacy (β = 0.14, *p* < 0.001) and student satisfaction (β = 0.06, *p* < 0.001), the associations were fairly small (< 0.20) (see Figure 1).

## Discussion

### Students’ Perceptions of Facilitation Strategies, Student Learning Motivation, and Satisfaction

The results indicated that most university students appreciated the facilitation strategies adopted by instructors in the cloud-based virtual classroom in mainland China. Students agreed that they had a certain amount of interaction with their instructors and peers and that instructors introduced innovations in their online courses. In addition, the students agreed that they had a strong inner drive and confidence in performing learning tasks and were moderately satisfied with the classes implemented in a synchronous online learning environment. Previous studies indicated that instructors providing timely responses to students and feedback on their questions were the most helpful facilitation strategies for acquiring the proper knowledge in synchronous online learning settings (Martin et al., 2018, 2019). Instructors have adopted various learning tools that allow real-time knowledge sharing and timely responses to create a satisfactory learning environment and enhance students’ behavioral and cognitive engagement via instructor scaffolding for interaction (Kuo et al., 2014).

In this study, students reported higher levels of affect/emotion than self-efficacy and satisfaction. This finding differs from the results of Lim and Kim’s (2003) study, which showed a higher level of learning self-efficacy than affect/emotion in a blended online learning setting. This discrepancy is probably due to the lack of collaborative learning and action-oriented learning opportunities, such as student collaboration projects, in synchronous online learning environments (Kuo et al., 2014), which are critical for meaningful learning and transfer (Crook, 1994). However, a recent study indicated that the proper use of scaffolding cloud tools has the potential to support collaborative learning in synchronous online learning environments (Baanqud et al., 2020).

This study revealed no significant difference in perceptions of instructors’ facilitation strategies and their learning outcomes among students with different demographic characteristics. Current evidence on the role of demographic characteristics in students’ online learning outcomes is conflicting and elusive (Dang et al., 2016). The influence of demographic characteristics on students’ online learning success could be affected by many contextual factors, such as the social culture and the applied operating principles of online learning systems (Cole et al., 2014; Harvey et al., 2017). Therefore, more studies should be conducted in synchronous online learning environments in the future.

### The Relationship Between Facilitation Strategies and Learning Outcomes

The SEM results provide empirical evidence that students’ perceptions of instructor-student interaction and instructor innovation facilitate their affect/emotion and self-efficacy. These relationships highlight the need to develop relevant strategies for enhancing self-efficacy in the context of synchronous online learning for better educational outcomes (Lazowski and Hulleman, 2015). A previous study indicated that students’ self-efficacy develops through positive learning experiences (Parschau et al., 2013). Therefore, the positive influence of instructor-student interactive scaffolding, a key element of online learning environments (Baanqud et al., 2020), on students’ academic and Internet efficacy might help to create positive learning experiences in synchronous online settings. Similarly, as previous studies have suggested that university students’ self-efficacy can be enhanced through course design intervention in asynchronous online settings (Fritea and Opre, 2015), instructor innovation, e.g., diversified technological methods for teaching, could also be effective for enhancing students’ self-efficacy in synchronous virtual classrooms. Therefore, instructors should consider how to make maximum the use of synchronous online technological tools to establish a novel and effective online learning environment.

Our results also indicate that students’ perceptions of instructor-student interaction and instructor innovation influenced their affect and emotion. This result is consistent with the findings of an earlier study that indicated that instructor presence was positively related to student emotion/affect in blended learning environments (Law et al., 2019). Students need to be actively involved in the learning process through interacting with their instructors and others, such as the learning content and their peers. Moreover, our results show that instructor innovation has a prominent effect on affect/emotion in a cloud-based virtual classroom, and this is consistent with previous evidence of this effect in asynchronous online settings (Lee, 2011). It should also be noted that the effect size of instructor innovation was larger than that of instructor-student interaction in the prediction of student affect/emotion in this study. According to the community of inquiry model (Garrison et al., 2000), emotions expressed in the online context exist in social, cognitive, and teaching presence. Instructor innovation, as an organizing element of teaching presence, is critical in providing socio-emotional support in cloud-based virtual classrooms. Although a previous study suggested that providing socio-emotional support is easier in the face-to-face environment (Garrison, 2007), our study suggests that face-to-face dialectical conversation in classroom settings could be closely simulated by the adoption of technological innovations supported by cloud computing and service technology (Al-Samarraie and Saeed, 2018).

Our results are also consistent with the findings of many previous studies that have revealed positive relationships between instructor-student interaction and student satisfaction in synchronous online learning settings (Kuo et al., 2014), and between instructor innovation and learning satisfaction in asynchronous online settings (Lee, 2011). Social presence theorists believe that instructors’ highly immediate behaviors lead to changes in students’ attitudes, including satisfaction (Richardson and Swan, 2003). Accordingly, with the availability of some functional features that students favor most (e.g., bullet subtitles and instant discussion boards), instructors are more likely to show immediate behaviors that improve communications with students and increase their satisfaction. However, the effect size of instructor-student interaction was larger than that of instructor innovation in the prediction of student satisfaction; this was probably due to the lack of instructors’ online teaching experience in a pure synchronous learning environment. The majority of instructors had very little experience of online teaching and using synchronous online learning tools, and thus were less likely to adopt technological innovation to create a more successful learning environment.

In contrast with the findings of a previous study that indicated that student interaction was a significant predictor of student satisfaction in synchronous online settings (Kuo et al., 2014), in our study, the relationships between student interaction and their perceived learning motivations and satisfaction were not significant. This difference is probably due to the lack of student collaboration projects in teaching goal orientation in synchronous online learning settings. Chinese students prefer teacher-centered learning in which knowledge is imparted directly from the instructor (Levinsohn, 2007), which may result in more active instructor-student interaction than student interaction.

### Limitations and Directions for Future Research

This study provides insights into the relationship between university students’ perceptions of instructors’ facilitation strategies and their learning outcomes in synchronous online learning environments. Some limitations should be noted as directions for future research. First, the study was based on data collected three months after the onset of synchronous online learning. Such a short implementation period may have limited the interactions between students and attenuated the potential influence of student interactions on their learning motivation and satisfaction. Meanwhile, due to the nature of the COVID-19 lockdown, students’ expectations of instructors’ teaching quality and methods might have been different from such expectations when more options are available. Therefore, comparative studies may be conducted across different learning environments in the future to confirm the findings of this study. Second, due to the cross-sectional design of this study, it was not possible to confirm consistent causal relationships between facilitation strategies, learning motivations, and satisfaction. In the further, longitudinal research would help to confirm causal relationships of these variables. Finally, the study sample consisted of university students from a province in eastern China, which may limit the generalizability of our findings. Future studies could consider recruiting participants with more diversified backgrounds.

## Conclusion and Implication for Practice

This study examined university students’ perceptions of facilitation strategies, learning motivation, and satisfaction, and the relationships between them in a cloud-based virtual classroom in mainland China. The results showed that students perceived high levels of facilitation strategies, learning motivation, and satisfaction. Students’ demographic characteristics, such as discipline, university type, gender, and grade, did not significantly affect their perceptions of facilitation strategies and learning outcomes. Instructor-student interaction and instructor innovation were found positively related to student learning motivation and satisfaction whereas the relationships between student interaction and learning motivation and satisfaction were weak and had no practical meaning. The findings of this study have implications for creating more effective synchronous online learning environments and achieving desirable learning outcomes. First, comprehensive technical training could be offered to facilitate instructors’ mastery of technologies in synchronous online learning to encourage them to implement pedagogical innovations. Second, considering the positive role of student interaction in stimulating favorable learning outcomes, instructors and administrators should consider encouraging more interactions among students in synchronous online learning environments. Thus, collaborative learning and task-based learning activities might be involved in the design of synchronous online learning courses. Third, the significance of instructor-student interaction implies that instructors could further enhance effective interactions with students through the use of quantitative data collected by synchronous online learning applications, for which cloud computing techniques allow students’ anonymous participation. This kind of quantified feedback makes it possible for instructors to identify which parts of the teaching content need further explanation.

## Data Availability Statement

The raw data supporting the conclusions of this article will be made available by the authors, without undue reservation.

## Ethics Statement

The studies involving human participants were reviewed and approved by Shandong University. The patients/participants provided their written informed consent to participate in this study.

## Author Contributions

RW and CG drafted the manuscript. JH and CL revised and proofread the manuscript. All authors contributed to the study design and approved the final version of the manuscript for submission.

## Conflict of Interest

The authors declare that the research was conducted in the absence of any commercial or financial relationships that could be construed as a potential conflict of interest.

## Publisher’s Note

All claims expressed in this article are solely those of the authors and do not necessarily represent those of their affiliated organizations, or those of the publisher, the editors and the reviewers. Any product that may be evaluated in this article, or claim that may be made by its manufacturer, is not guaranteed or endorsed by the publisher.
